# The effects of cognitive behavioral therapy-based digital therapeutic intervention on patients with alcohol use disorder

**DOI:** 10.3389/fpsyt.2025.1486338

**Published:** 2025-06-09

**Authors:** Song-Hee Lim, Jae-Kyoung Shin, Moo Eob Ahn, Chang-hyun Lee, Sang-Kyu Lee

**Affiliations:** Chuncheon Sacred Heart Hospital, Hallym University, Chuncheon, Republic of Korea

**Keywords:** digital therapeutic intervention, alcohol use disorder, cognitive behavioral therapy (CBT), digital therapeutic CBT, addiction treatment

## Abstract

**Introduction:**

This study investigated the effectiveness of a digital therapeutic intervention for individuals with alcohol use problems. Digital interventions are increasingly considered viable alternatives or complements to traditional face-to-face treatments, especially in improving accessibility and adherence.

**Methods:**

A total of 30 outpatients were recruited and randomly assigned to either a digital intervention group or a face-to-face cognitive behavioral therapy (CBT) control group. After excluding two dropouts, data from 28 participants were analyzed. The digital intervention group received a 12-week mobile application-based CBT program, which included 84 video-based CBT sessions. The control group received an 8-session standardized face-to-face CBT program over the same period. Both groups were also provided with a mobile application that included a diary feature for tracking alcohol consumption, cravings, and mood. Assessments were conducted at baseline, mid-treatment (week 4), and post-treatment (week 12) to evaluate risky drinking, craving, readiness for change, depression, anxiety, and alcohol-related symptoms.

**Results:**

The primary outcome, abstinence rate during weeks 9–12, was significantly higher in the digital intervention group (73.3%) compared to the control group (30.8%). Regarding secondary outcomes, the digital group showed significantly greater reductions in risky drinking, craving, and anxiety levels compared to the control group. However, no significant group differences were found for other outcome variables.

**Discussion:**

These findings suggest that digital CBT-based interventions can be an effective alternative to face-to-face CBT for reducing alcohol use and related symptoms. The study highlights the potential of digital therapeutics in addiction treatment, while acknowledging limitations such as small sample size and short follow-up period. Future research should explore long-term effects and broader clinical applicability.

**Clinical trial registration:**

https://cris.nih.go.kr/cris/search/detailSearch.do?seq=29717&status=5&seq_group=29562&search_page=M, identifier KCT0010289.

## Introduction

Alcohol use disorder (AUD) refers to the continued consumption of alcohol despite persistent physical, psychological, and interpersonal problems caused by alcohol use. Alcohol dependence is characterized by clinically significant behavioral and psychological changes that occur during or shortly after alcohol consumption. According to the Diagnostic and Statistical Manual of Mental Disorders, Fifth Edition (DSM-5) (1), it is essential to approach alcohol-related issues along a continuum. The DSM-5 emphasizes the need to integrate alcohol dependence and alcohol abuse into a single dimension known as alcohol use disorder, based on research findings that indicate these conditions are part of a single spectrum.

AUD is a chronic and highly recurrent condition, recognized not just as an individual problem but as a severe social issue. Studies indicate that over 280 million people worldwide are affected by AUD, which accounts for approximately 4.1% of the global adult population (2). Furthermore, alcohol-related mortality is a significant public health concern globally, According to reports from WHO in 2016 about 3 million people dying annually due to alcohol-related causes, representing around 5.3% of all deaths worldwide (3). These statistics may vary depending on cultural, economic, and social factors in different countries.

As of 2016, the prevalence of AUD in South Korea was 6.2%, which is approximately 1.7 times higher than the Southeast Asian average of 3.9% (4). Specifically, according to the 2021 Korean National Mental Health Survey, the lifetime prevalence of AUD in South Korea was 11.6%, the highest among major mental disorders (5). This high prevalence is closely linked to the permissive drinking culture in Korea.

In a 2016 United Nations survey on annual per capita alcohol consumption, South Korea ranked 17th out of 124 countries, placing it in the top 15% of alcohol consumption globally. Among major countries, it ranked just below Russia (13.9 liters) and the United Kingdom (12.3 liters). Furthermore, over five years, South Korea showed the highest alcohol consumption among four Asian countries in the Organisation for Economic Co-operation and Development (OECD), surpassing major OECD countries such as the United States, Japan, and the United Kingdom, excluding key European nations like Germany and France (6).

Korea’s drinking culture is characterized by easy access to alcohol, a permissive attitude towards drinking, and a widespread tendency to encourage excessive drinking or tolerate heavy drinking behaviors (7). However, there is a rare tendency to view alcohol-related disorders as psychiatric problems or diseases, leading to a high incidence of alcohol disorders and a vulnerability in addressing them.

Alcohol-related issues are particularly challenging due to their high prevalence and relapse rates. Among those with alcohol dependence, 71.5% to 82.1% relapse within 3 to 4 months, and 65% to 90% resume drinking within one year after treatment (8). In South Korea, the relapse rate for alcohol dependence within six months ranges from 44.5% to 80.3% (9).

To address the severe and long-term problems caused by alcohol dependence, treatment efforts involve the application of multiple treatment programs to enhance recovery rates. These include combining biological vulnerability treatments with psychosocial interventions such as cognitive-behavioral therapy (CBT), stages of change therapy, relapse prevention, motivational interviewing, exposure-response prevention techniques, and social support network therapy (10–14).

The paradigm in mental health has shifted from post-treatment to recovery-oriented approaches, leading to changes in the clinical field of alcohol dependence treatment. In the Korean version of the 2011 clinical guidelines for AUD published by the Korean Society for Addiction Psychiatry, various psychosocial treatment techniques, including Motivational Enhancement Therapy (MET) and CBT, are strongly recommended with the highest grade of recommendation (Grade A). CBT, in particular, is known to produce the best treatment outcomes when combined with medication (15).

CBT is based on cognitive theory, which posits that emotions and behaviors are influenced by one’s thoughts. It involves identifying automatic thoughts, restructuring cognitive distortions that trigger negative emotions into more rational and adaptive thinking, and promoting positive changes. Key components commonly used in CBT include collaboration, case conceptualization, structured therapy sessions, cognitive and motivational strategies, continuity of care, cue exposure, psychoeducation, and coping skills training (16).

While traditional treatment methods are effective, the advancement of digital technology opens new possibilities for complementing and enhancing these approaches. In mental health, digital therapeutic interventions combined with traditional therapies like CBT can play a significant role in improving patients’ treatment experiences.

Digital therapeutic interventions are innovative approaches that use digital technology to address various health issues. Digital addiction treatment allows patients to receive treatment via web or mobile applications without needing to visit a hospital, helping to reduce substance use. Because therapeutic training in daily life is effective in addiction treatment, digital interventions can enhance accessibility, reduce spatial and temporal constraints, and improve treatment effectiveness (17).

Moreover, digital addiction treatment allows for the development of personalized treatment plans based on continuously monitored data. Digital therapy is actively applied in clinical settings, with recent studies exploring digital treatments for opioid and alcohol addiction.

For example, a clinical trial was conducted to verify the effectiveness of the digital therapeutic device reSET-O for opioid addiction. In this study, 170 patients were randomly assigned to a single-blind trial (18). The control group received standard treatment, while the experimental group used a digital therapeutic device containing 67 modules alongside standard treatment. Over 12 weeks, the experimental group showed significantly higher treatment retention and abstinence rates between weeks 9 and 12 compared to the control group.

In alcohol addiction treatment, the “Drink Less” program, developed based on Medical Research Council (MRC) guidelines and the Multiphase Optimization Strategy (MOST), showed promising results (19). An exploratory clinical trial conducted over four weeks found that an enhanced version of the normative feedback and cognitive bias retraining modules significantly affected alcohol consumption changes. Additionally, an enhanced version of the self-monitoring and feedback and action planning modules significantly impacted the total score of the Alcohol Use Disorders Identification Test (AUDIT). While these modules are important for optimizing alcohol addiction treatment, additional confirmatory clinical trials are needed to compare their effectiveness with standard treatment.

Another preliminary study on digital alcohol addiction treatment involved “Vorvida,” an internet-based treatment program (20). In this study, the experimental group received treatment through the Vorvida app for three months, while the control group received standard treatment or was placed on a waiting list. Over time, the experimental group showed significant reductions in monthly alcohol consumption (QFI) and Timeline Followback (TLFB) indicators compared to the control group.

Although research on digital addiction treatment is increasing, some controversy remains regarding its effectiveness. Considering various limitations, more research is needed to verify the effectiveness of digital addiction treatment, with a number of studies comparable to those validating the effectiveness of standard treatment. However, the number of studies verifying the effectiveness of digital addiction treatment remains limited.

Building on this background, the present study aims to investigate the effects of digital therapeutic intervention on alcohol addiction by comparing a group that received digital intervention therapy with a control group that received traditional face-to-face cognitive behavioral therapy.

## Methods

### Study design

#### Participants

Participants for this study were recruited from outpatient clinics. The inclusion criteria mandated that all participants were adults aged 19 years or older who had a pre-existing clinical diagnosis of either: (1) Alcohol Use Disorder (AUD) according to the DSM-5 criteria, or (2) mental and behavioral disorders due to alcohol use (F10) as defined by the ICD-10.

Specifically, these diagnoses were established by a qualified psychiatrist through a comprehensive clinical evaluation, adhering strictly to the diagnostic criteria outlined in the DSM-5 and ICD-10.

Participants were required to be proficient in reading and writing in Korean and to be able to use mobile applications on common devices such as smartphones or tablet PCs. They needed to provide written informed consent after receiving a thorough explanation of the clinical trial, understanding its purpose, and agreeing to comply with the study procedures. Additionally, participants were excluded if they were diagnosed with dementia as determined by a clinical physician.

The study excluded participants who met the following criteria. Individuals with severe or progressive diseases that could pose a risk to their health were not included. Additionally, those with medical or neurological conditions that may lead to cognitive impairment, such as cerebral palsy, encephalitis, or meningitis, as well as individuals diagnosed with psychotic disorders like schizophrenia, were also excluded.

Furthermore, individuals who had experienced a traumatic brain injury within the past three years, resulting in a loss of consciousness for more than one hour or requiring hospitalization, were not eligible. Those with significant hearing or vision impairments that could interfere with clinical trial procedures were also excluded. Lastly, individuals deemed unsuitable for the study based on the researcher’s assessment were not included.

A total of 30 participants met the inclusion criteria and were enrolled in the study. During the study, 1 male and 1 female participant dropped out, resulting in a final analysis that included 20 males and 8 females. The reason for dropout was voluntary withdrawal from the study. Among the 28 participants included in the final analysis, 20 were male (67.86%) and 8 were female (32.14%). The mean age of the participants was 47.43 years (SD = 9.27), and the age range was 23 to 65 years. The mean age by gender was 46.47 years (SD = 10.28) for males and 49.44 years (SD = 6.73) for females, with age ranges of 23 to 65 years for males and 37 to 58 years for females.

### Study procedures

Participants provided informed consent after a detailed explanation of the study, and were then evaluated for eligibility based on selection and exclusion criteria.

Random assignment was conducted for participants who met all selection and exclusion criteria, assigning them to either the experimental group or the control group.

Prior to receiving treatment, participants completed self-report questionnaires to assess risky drinking, cravings, readiness for change, depression, anxiety, and alcohol-related symptoms.

Participants installed and logged into the application using an access code provided through a web program. The investigator explained to the participants in the experimental group how to use the application and to the subjects in the control group, the procedures related to daily check.

The experimental group participated in digital cognitive behavioral therapy (CBT) program for alcohol use disorder treatment daily for 84 days, and the results were automatically provided through a web program accessible to healthcare providers. Participants used the diary and notification functions to record their daily abstinence status, craving levels, and mood.

The control group installed an application that did not include CBT-based treatment content and was provided only with diary and notification functions to record their daily abstinence status, craving levels, and mood for the same period. In addition, the control group underwent 40–50 minute CBT sessions every two weeks, with a total of seven in-person sessions.

Both groups received treatment for the same 12-week period and completed self-report questionnaires at the start of treatment, at the 4-week mark, and at the end of the 12-week period.

The self-report questionnaires administered at baseline, 4 weeks later, and at the end of the intervention included not only the AUQ-K to measure alcohol cravings but also the AUDIT-K, RCQ, CIWA-Ar, HADS, CRQ, BADS, MAAS, SOCRATES-K, and HAIS. After the baseline measurement, AUDIT-K, AUQ-K, HADS, and HAIS were administered at both the 4-week and post-intervention time points.

**Figure 1:**
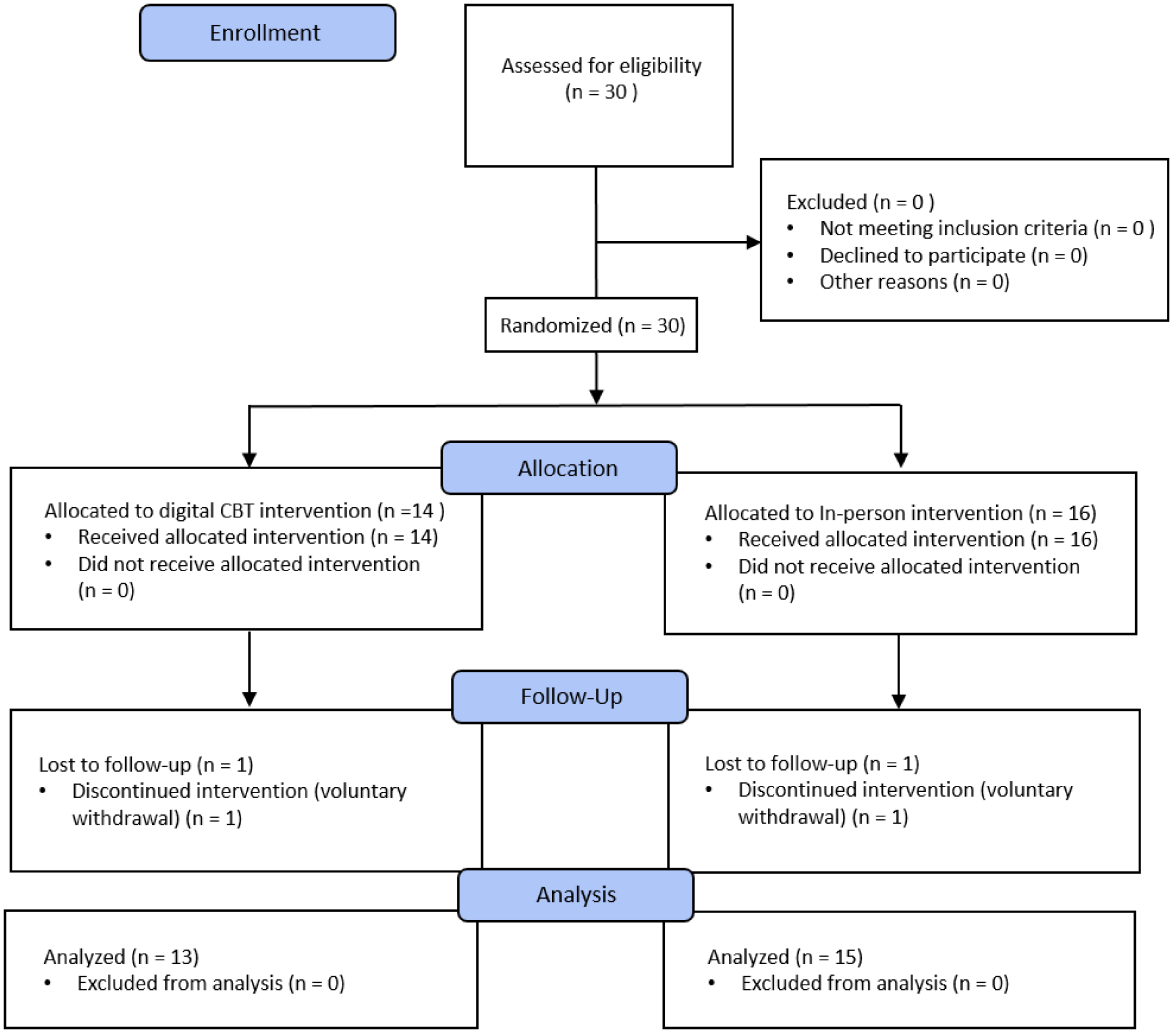
Flowchart of participant enrollment, randomization, and treatment.

**Figure 1** shows the flow of participant allocation and treatment procedures throughout the study.

#### Randomization procedure

An access code is generated in the clinician’s web program for patients who meet all the inclusion and exclusion criteria. Then, patients are randomly assigned using the Interactive Web Response System (IWRS) into either the experimental group, which receives the CBT-based digital therapeutic intervention, or the control group, which receives traditional cognitive-behavioral therapy.

#### Blinding procedure

A single-blind design is implemented, where the control group, receiving face-to-face cognitive-behavioral therapy, is also provided with an app that does not include digital CBT content but only contains a diary feature (for tracking abstinence status, craving levels, mood, etc.) and notification functions.

### Intervention

#### Cognitive behavioral therapy-based digital therapeutic intervention

The cognitive-behavioral therapy based digital therapeutic intervention for patients with alcohol use disorder is designed to treat symptoms of alcohol use disorder in patients suffering from mental and behavioral disorders caused by alcohol use, utilizing a cognitive-behavioral approach. This intervention consists of a web-based program called the “Clinician Dashboard,” which helps healthcare providers effectively monitor and manage patients’ treatment progress, and a patient application software that supports patients in actively participating in their own treatment.

Patients receiving the digital therapeutic intervention are provided with a total of 84 treatment modules over a 12-week period and are encouraged to complete one module each day. Each treatment module consists of a pair, including a video and a worksheet, designed so that patients can complete the worksheet after watching the video. Additionally, patients are allowed to revisit and complete any missed modules without limitation, but they are restricted from accessing future modules in advance.

The video component of each treatment module is specifically designed to correct maladaptive thoughts and cognitive errors that perpetuate alcohol use disorder, and to prevent the chronic progression of alcohol use disorder. The other component of the treatment module, the worksheet, is a tool designed to help modify maladaptive thoughts and behaviors related to alcohol use. It includes activities such as thought recording, behavior planning, and goal setting. This design enables patients to systematically record and analyze their thoughts, emotions, and behaviors. In addition to CBT techniques, the treatment modules incorporate various therapeutic elements aimed at reducing resistance to treatment and helping patients discover their own motivation for recovery. These elements include motivational enhancement therapy, psychoeducational interventions, mindfulness for managing cravings, and relapse prevention strategies.

The digital therapeutic intervention group is provided with the following features, identical to those offered to the control group. First, participants can use a simple checkbox to record whether or not they consumed alcohol each day. This sobriety tracking feature helps users monitor their progress and see how long they have remained alcohol-free. Additionally, healthcare providers can monitor these records through the web-based program. Secondly, participants have access to a craving tracking feature, which allows them to record the intensity of their alcohol cravings. Alongside this, they can also record their daily emotions, their motivation to remain sober, and the perceived likelihood of drinking, all measured on a 5-point Likert scale, except for the sobriety tracking. Participants are also supported with a step-tracking feature that allows them to monitor their physical activity. Lastly, the system calculates a comprehensive health index based on all these data points and displays it as a graph on the home screen, enabling participants to visually understand their overall health status. Additionally, the system graphs the changes in cravings, mood, and sobriety over time, helping users identify correlations between their emotional state and cravings.

#### Cognitive behavioral therapy for alcohol use disorder (control group)

The control group received face-to-face cognitive behavioral therapy (CBT) intervention without digital therapeutic intervention for a duration of 12 weeks. This face-to-face CBT consisted of a total of 7 sessions, each lasting 40 to 50 minutes. The content of the sessions included preparation before therapy, exploration of drinking episodes, completion of drinking outcome records, managing negative emotions, coping with alcohol cravings and urges, problem-solving and goal setting, techniques for refusing alcohol, and relapse prevention.

The control group was also provided with an application that included only a sobriety diary feature (tracking sobriety status, cravings, and mood) and notification functions, without the digital CBT modules. Through this application, the control group was able to record and monitor their sobriety status throughout the treatment process.

The therapist who provided CBT at the hospital was one person, and this therapist was a clinical psychology resident who had completed a master’s degree in clinical psychology. The therapist received training and supervision from Clinical Psychologist and a psychiatrist, and adhered to the therapist manual while conducting the therapy.

### Outcome measurement

#### Cognitive behavioral therapy-based digital therapeutic intervention

##### Mobile application

During the treatment period, participants recorded their alcohol use using the application. The application feature allows patients to record their alcohol use through a button, which helps measure their abstinence status and duration. Evaluators can check the abstinence status and the length of abstinence in real-time using data collected from participants through a web program designed for healthcare providers. This enables the assessment of whether participants maintained abstinence throughout the total 84-day treatment period and during the assessment period between weeks 9 and 12.

### Self-report measures

#### Korean version of Alcohol Use Disorder Identification Test

The AUDIT-K used in this study is the Korean version of the Alcohol Use Disorder Identification Test (AUDIT), developed by the WHO. The AUDIT-K is a tool designed to measure alcohol consumption levels, frequency, quantity, symptoms of dependence, and alcohol-related problems. It consists of 10 items, with each item being rated on a scale of 3 to 5 points. Originally, the assessment period was set for one year, but for this study, it was applied with a two-week interval.

The outcome measure is calculated based on the responses provided by the participants to the AUDIT-K items, with each item scored individually to compute a total score. This score is treated as a continuous variable and is used to evaluate the level of alcohol-related risk. The results for each item are coded as continuous variables, with individual item scores ranging from 3 to 5 points. The total score is calculated as a continuous variable, ranging from a minimum of 0 to a maximum of 40 points.

#### Readiness To Change Questionnaire

The Readiness to Change Questionnaire (RCQ) is a tool developed to measure the treatment motivation of individuals with alcohol dependence (21). This assessment helps determine which stage of change—Precontemplation (P), Contemplation (C), or Action (A) a person is in during their recovery process. The RCQ consists of 12 items, with 4 items for each subscale, and the stage with the highest score is identified as the individual’s current stage of readiness for change. In Korea, Yoo adapted this tool (22), and subsequent reliability testing was conducted by Taekyung Lee and colleagues to ensure its applicability in the Korean context.

#### Korean version of Alcohol Urge Questionnaire

The Alcohol Urge Questionnaire (AUQ) was developed as a self-report tool to measure a single factor of alcohol craving, specifically designed for research on alcohol-related urges (23). The questionnaire consists of 8 items, each rated on a 7-point Likert scale ranging from 0 to 6, with a higher score indicating a greater craving for alcohol. In Korea, Kim et al. (24) validated the reliability of this tool. The total score of this questionnaire, as a continuous variable, was used to measure the level of alcohol craving.

#### Clinical Institute Withdrawal Assessment for Alcohol Scale

The Clinical Institute Withdrawal Assessment of Alcohol Scale, Revised (CIWA-Ar) is a scale developed by Sullivan et al. to measure the severity of withdrawal symptoms. It consists of 10 items, including nausea and vomiting, tremor, sweating, anxiety, auditory disturbances, visual disturbances, tactile disturbances, headache, agitation, and orientation and clouding of sensorium. All items, except for disorientation (rated 0-4), are rated on a scale of 0-7, with a total score range of 0–67 to assess the severity of withdrawal symptoms.

#### Hospital Anxiety and Depression Scale

The Hospital Anxiety and Depression Scale (HADS) is a 14-item assessment tool developed by Zigmond and Snaith (25) to measure the levels of anxiety and depression specifically in hospital settings. The scale is structured such that the 7 odd-numbered items assess anxiety, while the 7 even-numbered items evaluate depression. In Korea, Min and colleagues translated and standardized the HADS in 1999, ensuring its applicability and cultural relevance for Korean patients (26). The total score of the odd-numbered items of this questionnaire was used to assess the level of anxiety. Additionally, the total score of the even-numbered items was used to assess the level of depression.

#### Cognitive Reappraisal Questionnaire

To measure the level of cognitive reappraisal, the Cognitive Reappraisal Questionnaire (CRQ), developed and validated by Kim Yoon-kyung in 2019, was used. This tool is designed to assess two subtypes of cognitive reappraisal. First, the objective reappraisal measures the ability to view situations neutrally and objectively, maintaining emotional distance during the evaluation of emotional experiences. The positive reappraisal, on the other hand, assesses the ability to shift focus towards the positive aspects of a situation and the potential benefits that can be gained by overcoming it. The CRQ consists of 20 items, and in Kim Yoon-kyung’s study, the internal consistency, as measured by Cronbach’s α, was found to be high. Specifically, the reliability of the CRQ was reported with a Cronbach’s α of.88 for the objective reappraisal items and.94 for the positive reappraisal items, indicating strong reliability and consistency (27).

#### Korean version of the Behavioral Activation for Depression Scale

The Behavioral Activation for Depression Scale (BADS) was developed by Kanter, Mulick, Busch, Berlin, and Martell to more accurately assess the level of patient activation and treatment response (28). In this study, the BADS was used to measure the level of behavioral activation. This scale consists of 25 self-report items and is effective in evaluating the outcomes of therapeutic interventions throughout the behavioral activation treatment process. The BADS is composed of four subscales: Activation, Work/School Impairment, Social Impairment, and Avoidance/Rumination. In Korea, the Korean version of the BADS was standardized by Oh Ji-hye and colleagues in 2017 (29).

#### Korean version of Mindfulness Attention Awareness Scale

To measure mindfulness attention and awareness, the Mindfulness Attention Awareness Scale (MAAS) developed by Brown and Ryan (30) was used. This scale primarily assesses inattentiveness or lack of awareness experienced in everyday life. It consists of 15 items, with participants rating the frequency of experiencing situations related to each item on a 6-point scale ranging from 1 (Almost Always) to 6 (Almost Never). The Korean version of MAAS, a Korean translation of the MAAS developed by Brown and Ryan, was used in this study. The reliability and validity of the K-MAAS were verified by Kwon and Kim (31).

#### Korean version of Stages of Change Readiness and Treatment Eagerness Scale

The Stages of Change Readiness and Treatment Eagerness Scale (SOCRATES) was developed to assess the motivation for change in problem drinkers (32). The scale is designed with four subscales: Precontemplation, Contemplation, Determination, and Action stages. It consists of 19 items, each rated on a 5-point Likert scale. Chun Young-min (33) validated the reliability and validity of this scale and developed the Korean version, SOCRATES-K.

#### Hanil Alcohol Insight Scale

To measure the level of insight in patients with alcoholism, the Insight Evaluation Scale developed by Kim et al. (34) was used in a clinical setting. This scale consists of 20 items, including both positive and negative questions, and utilizes a 3-point scale. It assesses awareness of one’s own drinking, recognition of loss of control over drinking and dependence, acknowledgment of the need for abstinence, understanding that one’s drinking contributes to current problems and causes distress to others, and the necessity of treatment. Higher scores indicate a greater level of insight into one’s condition. The total score of this questionnaire was used to measure the overall illness.

### Statistical analysis

Repeated measures ANOVA was conducted to analyze the differences in change amounts between groups. The analysis was performed by setting the groups (experimental group, control group) as between-subject factors and the measurement times (pre, post) as within-subject factors. To evaluate the differences between groups in terms of abstinence performance, the differences in total days of abstinence between groups were examined using an independent-sample t-test. Abstinence success was defined as not drinking even once between weeks 9 and 12, and the success rates between groups were analyzed using Pearson’s chi-square test. Moreover, to examine the interaction effects of groups and measurement times on each dependent variable, a repeated measures ANOVA was performed by setting the groups (experimental group, control group) as between-subject factors and the measurement times (pre, post) as within-subject factors. Log data analysis was conducted to examine the differences between groups in total number of accesses, total access days, total usage time (minutes), average daily app launches, and app usage time (minutes). Additionally, a regression analysis based on log data was conducted to explore the differences between groups in greater detail. Given the small sample size, effect sizes (Cohen’s d) were also calculated to assess the magnitude of differences between groups in the relevant measures.

## Results

### Comparison of digital therapy usage between experimental and control groups

The usage comparison between the groups is summarized as follows. First, we assessed the extent to which patients in the experimental group utilized the cognitive-behavioral therapy-based digital alcohol treatment device.

In the experimental group, the average login frequency was 110.33, the average number of days of access was 40.67, and the average total usage time was 1084.80 minutes. Additionally, the average viewing time of the digital CBT modules was 195.20 minutes, the average number of completed worksheets was 34, and the average number of completed modules was 36.26.

In the control group, the average number of logins was 136.23, the average number of days of access was 45.54 days, and the average total usage time was 230.62 minutes. Additionally, the average number of completed CBT sessions was 36.26 (**Table 1**).

**Table 1:** Summary of user engagement and completion rates for digital intervention group and control groups.

Characteristic	Digital Intervention Group	Control Group
login frequency	110.33	136.23
Days of access(days)	40.67	45.54
Usage Time (min)	1084.80	230.62
Viewing Time (min)	195.20	N/A
Worksheet Completions(sessions)	34	N/A
Viewing Count(sessions)	36.26	N/A
Face-to-Face CBT Sessions	N/A	4.23

### Pretest equivalence test

To ensure that the treatment and control groups were comparable at baseline, a pretest equivalence test was conducted. The pretest scores for both groups were compared using an independent samples t-test. The results revealed no significant differences for any of the variables (see **Table 2**), indicating that the two groups were statistically equivalent before the intervention.

**Table 2:** Independent samples t-test for mean differences between the intervention and control groups.

Variable	Digital Intervention Group (n=13)		Control Group (n=15)		t-value
	Mean	SD	Mean	SD	
AUDIT-K	24.00	8.86	29.07	6.39	-1.712
RCQ	1.69	4.77	1.8	4.48	-0.061
AUQ-K	24.15	6.01	25.73	4.4	-0.783
CIWA-Ar	12.54	4.63	12.4	4.29	0.082
HADS -Anxiety	14.38	4.59	14.33	3.75	0.032
HADS -Depression	66.15	13.04	63.47	18.02	0.456
CRQ	67.77	24.79	68.4	20.59	-0.073
BADS	17.85	3.85	17.67	2.53	0.144
MAAS	62.85	6.9	62.33	7.76	0.185
SOCRATES-K	1.15	5.64	4.93	5.71	-1.758
HAIS	2.23	4.32	1.27	2.05	0.735

### Analysis of differences in change amounts between groups

To examine the effects of group (digital intervention group, control group) and measurement time (pre-test, post-test) on each dependent variable, a repeated measures ANOVA was conducted with group as the between-subjects variable and measurement time as the within-subjects variable (see **Table 3**).

**Table 3:** Repeated measures ANOVA results: interaction effect of group and time.

Dependent Variable	Group	Pre-Test (M(SD))	Post-Test (M(SD))	F
Risky drinking level (AUDIT-K Score)	Digital Intervention	29.63 (5.34)	3.63 (3.07)	10.146**
	Control	23.00 (4.66)	8.63 (7.17)	
Alcohol Craving (AUQ-K Score)	Digital Intervention	26.25 (2.82)	5.00 (3.30)	9.904**
	Control	24.13 (3.80)	12.63 (6.16)	
Anxiety (HADS-anxiety Score)	Digital Intervention	14.13 (2.53)	3.13 (3.68)	6.720*
	Control	11.75 (3.85)	7.00 (4.99)	
Depression (HADS-depression Score)	Digital Intervention	15.63 (3.07)	6.00 (5.71)	2.146
	Control	12.88 (4.70)	8.00 (2.73)	
Insight (HAIS Score)	Digital Intervention	5.50 (4.96)	10.63 (7.89)	0.030
	Control	0.88 (5.54)	5.25 (5.95)	

*p <.05, **p <.01.

**Figure 2:**
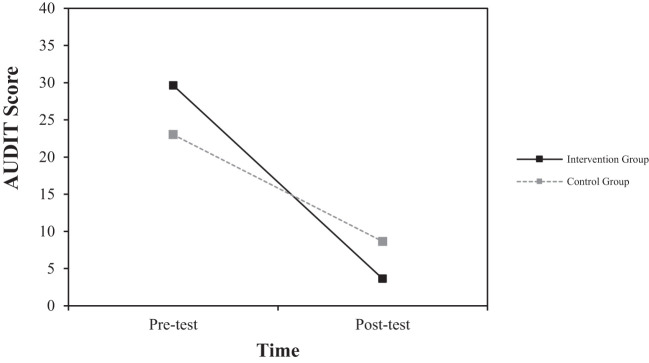
Interaction effect of group and time on changes in risky drinking levels as measured by AUDIT-K score.

First, the interaction effect between group (experimental vs. control) and time (pre-test vs. post-test) was significant [*F* (1, 14) = 10.146, *p* <.01]. As shown in **Figure 2**, the experimental group exhibited a larger decrease in risky drinking levels compared to the control group over the same period. This indicates that the experimental group experienced a more substantial reduction in alcohol use as measured by the AUDIT-K, suggesting that the intervention was effective in decreasing risky drinking behaviors.

**Figure 3:**
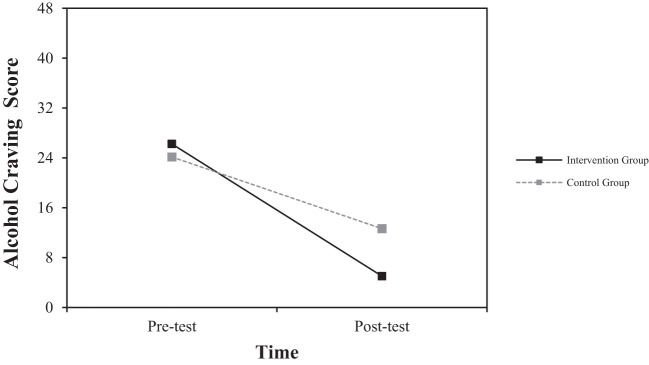
Interaction effect of group and time on changes in alcohol craving score.

Second, the interaction effect between group and time was significant for alcohol craving [*F* (1, 14) = 9.904, *p* <.01]. As shown in **Figure 3**, the magnitude of change from pre-test to post-test was greater in the experimental group compared to the control group. This indicates that the experimental group experienced a faster reduction in alcohol craving over the same period.

**Figure 4:**
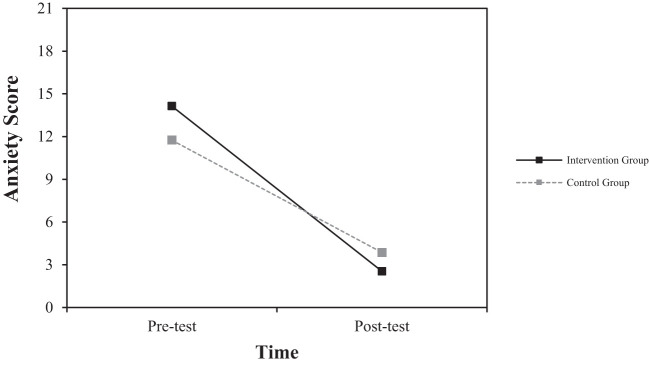
Interaction effect of group and time on changes in anxiety score.

Third, the interaction effect between group and time was also significant for anxiety [*F* (1, 14) = 6.720, *p* <.05]. As shown in **Figure 4**, the magnitude of change from pre-test to post-test was greater in the experimental group compared to the control group. This suggests that the experimental group experienced a faster reduction in anxiety over the same period. However, no significant group differences were observed for the remaining variables (Insight: *F* = 0.030; Depression: *F* = 2.146).

Based on the results of the repeated measures ANOVA, significant changes were observed in Risky drinking levels, Alcohol Craving, and Anxiety.

For Risky drinking levels, the digital intervention group showed a substantial decrease from 29.63 (5.34) at pre-test to 3.63 (3.07) at post-test, with a very large effect size of Cohen**’**s d = -5.97. In contrast, the control group showed a change from 23.00 (4.66) at pre-test to 8.63 (7.17) at post-test, with a moderate effect size of Cohen**’**s d = -2.38.

Significant changes were also observed in Alcohol Craving. The digital intervention group experienced a large decrease from 26.25 (2.82) at pre-test to 5.00 (3.30) at post-test, with a very large effect size of Cohen**’**s d = -9.93. The control group showed a change from 24.13 (3.80) at pre-test to 12.63 (6.16) at post-test, with a moderate effect size of Cohen**’**s d = -2.44.

Similarly, in Anxiety, the digital intervention group showed a significant decrease from 14.13 (2.53) at pre-test to 3.13 (3.68) at post-test, with a very large effect size of Cohen**’**s d = -3.48. The control group showed a change from 11.75 (3.85) at pre-test to 7.00 (4.99) at post-test, with a moderate effect size of Cohen**’**s d = -1.07.

### Differences between groups in terms of abstinence performance

#### Comparison of total abstinence days

An independent samples t-test was conducted to compare the total number of abstinence days between the groups. The control group had a mean of 73.15 days (SD = 10.915), while the experimental group had a mean of 79.20 days (SD = 8.495). The difference between the groups was not statistically significant (t = -1.617, p >.05).

#### Comparison of abstinence success rates

Pearson’s chi-square test was conducted to evaluate the abstinence success rates between the groups (see **Table 4**). The analysis revealed a significant difference in abstinence success rates between the groups (χ² = 5.073, p <.05). Specifically, the experimental group had a significantly higher abstinence success rate compared to the control group.

**Table 4:** Results of the abstinence success rate comparison between groups.

Group	Success	Failure	Success Rate (%)	χ²	*P*-value
Intervention	11	4	73.3	5.073	<.05
Control	4	9	30.8		

### Impact of log data metrics on post-intervention outcomes: a hierarchical multiple regression analysis

A hierarchical multiple regression analysis was conducted within the intervention group to investigate the predictive relationships between various log data metrics and post-intervention outcomes, including depression, anxiety, craving for alcohol, insight, and risky drinking levels. The analysis was structured as follows:

Step 1: A baseline model was established incorporating pre-measurement values as independent variables to provide a reference for evaluating the impact of subsequent variables.

Step 2: The model was then expanded by including additional predictors: total number of visits, total number of days visited, total usage time, and total viewing duration of intervention content. This allowed for the assessment of changes in explanatory power, highlighting the incremental value of these log data metrics in predicting post-intervention outcomes.

The results of the hierarchical multiple regression analysis, detailed in **Table 5**, showed that the total number of visits had a significant impact on the reduction of alcohol craving (β = -0.237, *p* = .029). Specifically, a higher number of visits was associated with a greater decrease in alcohol craving. In contrast, the total number of days visited did not significantly impact the reduction of alcohol craving and showed a tendency towards an increase (β = 0.313, *p* = .040). Other variables, including total usage time and total viewing duration of intervention content, did not have a significant impact on alcohol craving reduction. These findings suggest that the total number of visits plays a more crucial role in reducing alcohol craving compared to the total number of days visited.

**Table 5:** Hierarchical multiple regression analysis predicting reduction in alcohol craving based on log data metrics.

Model	Predictor Variables	*B*	*SE*	*β*	*t*	*p*
1	(Constant)	17.297	11.549			
	Pre-Measurement [AUQ-K Total Score]	-0.468	0.438	-0.400	-1.070	0.326
2	(Constant)	38.966	4.522		8.616	0.013
	Pre-Measurement [AUQ-K Total Score]	-0.896	0.136	-0.766	-6.587	0.022
	Total Number of Visits	-0.237	0.042	-2.273	-5.708	0.029
	Total Number of Days Visited	0.313	0.064	2.067	4.855	0.040
	Total Usage Time	0.008	0.002	2.020	3.299	0.081
	Total Viewing Duration of Intervention Content	-0.044	0.012	-2.516	-3.833	0.062

The unit for “Total Usage Time” and “Total Viewing Duration of Intervention Content” is minutes.

## Discussion

The role of digital therapeutic devices in the treatment of alcohol use disorder is becoming increasingly significant. These devices are evolving from a supplementary role—where they were used as adjuncts to traditional treatments—into a primary role within the treatment process. However, to fully evaluate the efficacy and effectiveness of these digital therapeutic devices, rigorous clinical trials and field validations are necessary.

This study aimed to determine whether the newly developed digital therapeutic approach provides therapeutic effects comparable to those of established face-to-face cognitive-behavioral therapy. To achieve this, we compared changes in alcohol use disorder-related symptoms between an experimental group receiving digital therapeutic interventions and a control group undergoing face-to-face cognitive-behavioral therapy.

The results of the pre-test equivalence evaluation for this pre-measurement showed no significant differences between the experimental and control groups, confirming the successful implementation of random assignment and indicating no statistically significant difference between the two groups.

The interaction effects of group and measurement time on each dependent variable were examined, and significant interaction effects were found for risky drinking levels, alcohol craving, and anxiety. This means that over the 12-week period from pre- to post-intervention, the changes in these three variables were significantly greater in the experimental group compared to the control group. Specifically, during the same period, the experimental group experienced a more rapid decrease in risky drinking levels, alcohol craving, and anxiety than the control group. The findings of this study suggest that digital intervention is not inferior to the cognitive-behavioral therapy, which has previously been proven effective.

To examine in detail, first, the experimental group experienced a faster reduction in risky drinking levels over the same period. These results suggest that digital therapeutic interventions may have a positive impact on effectively reducing risky drinking behaviors. The interactive elements and real-time feedback provided by the digital therapy could have contributed to the reduction in risky drinking, and the effectiveness of these digital interventions appears to be promising compared to traditional treatment methods.

Connecting these results to prior research, this study provides additional evidence supporting the effectiveness of digital interventions for alcohol use disorders. Previous studies have also demonstrated that digital interventions can effectively reduce various symptoms associated with alcohol use disorders. For example, a clinical study on the digital therapeutic device reSET-O for opioid use disorder involved 170 patients who were randomly assigned to either a control group receiving standard treatment or an experimental group receiving standard treatment plus a digital therapeutic device. Over a 12-week period, the experimental group showed significantly higher treatment retention and abstinence rates compared to the control group (35). These findings support the potential of digital interventions to effectively address substance use disorders. Additionally, a study utilizing the internet-based treatment Vorvida demonstrated that digital interventions significantly reduce alcohol consumption (20). This study compared a group of 608 adults with alcohol consumption issues who used the Vorvida app for 3 months with a control group that either received standard treatment or was on a waiting list. The results showed that the experimental group using Vorvida had a significantly greater reduction in alcohol consumption compared to the control group. These findings suggest that digital interventions can be an effective treatment option for alcohol use disorders.

Second, the Digital Intervention Group showed a faster reduction in alcohol cravings compared to the control group over the same period. Additionally, anxiety levels were also significantly reduced. This result suggests that digital therapeutic interventions may be more effective in reducing alcohol cravings and anxiety compared to standard treatment. Additionally, this finding supports the effectiveness of digital therapies as demonstrated by previous research. Prochaska et al. (36) reported that digital therapeutic interventions, such as the Woebot app, which utilizes artificial intelligence, led to significant improvements in alleviating and treating alcohol addiction symptoms. An 8-week preliminary clinical trial was conducted with 101 adults aged 18 to 65. The analysis of changes before and after treatment revealed that 86.1% of participants experienced approximately a 50% reduction in cravings for substances. Furthermore, confidence in overcoming substance urges significantly increased, and mental health indicators such as depression and anxiety also showed significant improvement.

To delve deeper into these significant findings, a thorough analysis of the log data from the experimental group was conducted. This analysis demonstrated that the frequency of visits positively impacted the reduction of alcohol cravings. Specifically, it was observed that more frequent participation in the intervention was associated with a greater decrease in cravings. This suggests that higher levels of user engagement are essential for maximizing the effectiveness of digital therapeutic interventions.

In digital therapy, the frequency of participation can play a crucial role in achieving effective outcomes. While consistent participation is also considered important in traditional face-to-face cognitive behavioral therapy (CBT), in digital therapy, how often a user participates can significantly influence the treatment’s effectiveness. In particular, because digital therapy involves user-driven participation, the frequency of participation is likely to be closely linked to the treatment outcomes. Such participation patterns act as an important variable in maximizing the effectiveness of digital therapy, highlighting a distinctive difference from traditional treatment methods.

Additionally, to prior research, user engagement appears to be a crucial factor in determining the success of digital therapies. A systematic review examining the impact of adherence and engagement on the effectiveness of e-therapy has emphasized the critical role of user engagement in the success of digital therapies (37). Similarly, research on internet-based interventions for anxiety and depression has highlighted that user engagement and adherence are key factors in achieving successful treatment outcomes (38).

However, it is important to note that not all studies agree on the direct correlation between user engagement and positive treatment outcomes. Mohr et al. (39) argue that high levels of user engagement do not necessarily guarantee better treatment results. They suggest that other factors, such as the content of the intervention, the user’s initial motivation, and the presence of external support, can significantly influence the effectiveness of the treatment.

Additionally, there is a notable gap in research specifically examining the impact of login frequency—how often patients access digital therapy programs—on treatment outcomes. This gap highlights the need for further research that considers these variables to better understand their influence on the success of digital therapies.

In addiction treatment, the frequency of addressing cravings is emphasized as being highly important. According to the study by Tiffany et al. (40), conducting treatment sessions frequently and continuously managing cravings helps to maximize the effectiveness of the treatment. Similarly, the research by Marlatt and Gordon (41) also highlights the importance of frequent treatment sessions and repeated interventions.

Based on these findings, the fact that the number of logins in this study affected the reduction of cravings suggests that understanding and utilizing login frequency metrics has significant implications for the development and improvement of effective digital therapeutic interventions. In particular, this study found that when separating the total number of logins and the number of days logged into the digital therapeutic app over a specific period, the number of logins had a more significant impact on reducing cravings.

Therefore, this study suggests that how often a user participates, rather than how consistently they participate, is a more critical factor in digital therapeutic interventions targeting alcohol use disorder. This finding underscores the importance of promoting regular and consistent use of digital therapeutic programs to achieve optimal treatment outcomes and provides crucial direction for setting participation metrics and adherence standards as digital health continues to evolve.

This study has several important limitations. First, instances where abstinence status was not confirmed were considered as abstinence. This approach reflects the concern that assuming unreported drinking as drinking could damage the trust between the patient and the therapist. However, assuming abstinence when drinking is not reported introduces a limitation in the accuracy of interpreting treatment outcomes.

Second, personalized modules were not provided. While digital-based addiction treatment allows for the development of detailed and tailored treatment plans based on continuously monitored data, and personalized treatment is generally more effective, this study applied the same intervention to all participants, which is a limitation.

Third, the sample size in this study was limited. The sample size was determined to assess whether the effects of CBT which has established efficacy, were comparable to those of CBT supplemented with digital interventions such as digital devices. However, due to the limited sample size, caution is required when interpreting and generalizing the results. Although there was a difference in AUDIT scores between the two groups at baseline, this difference may have been statistically significant if the sample size were larger. Future research should employ a larger sample size to strengthen the reliability of the results and allow for more robust statistical analysis. More importantly, efforts should be made to ensure the homogeneity of the two groups by applying rigorous randomization methods.

Fourth, a significant limitation of this study is that the actual use of the digital intervention tools by patients could not be directly verified. While digital interventions can contribute to improving treatment adherence and engagement, they cannot fully replace clinical treatment. This limitation may influence the interpretation of treatment outcomes. Furthermore, patients with depression were not excluded from this study, and there is a possibility that participants taking anxiolytics, antidepressants, or other medications were included. Therefore, the potential impact of medication use as a clinical variable affecting the efficacy of the digital intervention cannot be completely ruled out. Future studies should more precisely control for these variables and further enhance the validity of the results through analyses that consider various patient characteristics.

Fifth, in this study, all face-to-face CBT sessions in this study were conducted by a single therapist. While this approach enhanced the consistency of the intervention and allowed for close monitoring of treatment fidelity, it also introduces a potential limitation regarding therapist-specific effects. The use of a single therapist may limit the generalizability of the findings, as treatment outcomes could be influenced by the specific characteristics or clinical competence of that individual. In larger-scale studies, Including multiple therapists is a common practice to minimize such confounding effects. Future research should consider including more than one therapist or statistically controlling for therapist effects in order to increase the external validity of the results.

In summary, digital therapeutic interventions for alcohol use disorder, conducted with random assignments to ensure baseline equivalence, demonstrated superior effectiveness in abstinence success rates compared to face-to-face cognitive behavioral therapy (CBT). Additionally, reductions in hazardous drinking levels, craving, and anxiety were more pronounced in the experimental group than in the control group. However, most other variables did not show significant differences between the two groups. Given the small sample size and the single-institution nature of this study, these results require further validation through more rigorous methodologies. Nonetheless, the findings, which showed comparable results across most aspects and superior therapeutic efficacy in key variables such as abstinence success, craving, and hazardous drinking levels compared to face-to-face CBT, suggest that digital therapeutic approaches could be a promising intervention for patients with alcohol use disorder.

## Data Availability

The original contributions presented in the study are included in the article/supplementary material. Further inquiries can be directed to the corresponding author.
