# Correction: The effects of cognitive behavioral therapy-based digital therapeutic intervention on patients with alcohol use disorder

**DOI:** 10.3389/fpsyt.2025.1644936

**Published:** 2025-06-26

**Authors:** Song-Hee Lim, Jae-Kyoung Shin, Moo Eob Ahn, Chang-hyun Lee, Sang-Kyu Lee

**Affiliations:** Chuncheon Sacred Heart Hospital, Hallym University, Chuncheon, Republic of Korea

**Keywords:** digital therapeutic intervention, alcohol use disorder, cognitive behavioral therapy (CBT), digital therapeutic CBT, addiction treatment

In the published article, there was an error in the **Funding** statement. This statement previously said:

“The author(s) declare that financial support was received for the research and/or publication of this article. This research was supported in 2023 by the Korea Institute for Advancement of Technology (KIAT) and the Korea Health Industry Development Institute (KHIDI) (Project Nos. K_G012002295903, RS-2022-KH12584522182102840201).”

The corrected statement appears below:

“The author(s) declare that financial support was received for the research and/or publication of this article. This work was supported by the Technology Innovation Program (or Industrial Strategic Technology Development Program-Advanced Technology Center plus) (20022959, Development of a digital treatment device equipped with digital cognitive behavioral therapy (CBT) to improve alcohol use disorder) funded By the Ministry of Trade Industry & Energy(MOTIE, Korea); in addition, this work was supported by a grant of the Korea Health Technology R&D Project through the Korea Health Industry Development Institute (KHIDI), funded by the Ministry of Health & Welfare, Republic of Korea (grant number: HI22C0707, RS-2022-KH125845).”

The authors apologize for this error and state that this does not change the scientific conclusions of the article in any way. The original article has been updated.

